# *Leptospira fainei* Detected in Testicles and Epididymis of Wild Boar (*Sus scrofa*)

**DOI:** 10.3390/biology10030193

**Published:** 2021-03-04

**Authors:** Giovanni Cilia, Fabrizio Bertelloni, Domenico Cerri, Filippo Fratini

**Affiliations:** Department of Veterinary Sciences, University of Pisa, Viale delle Piagge 2, 56124 Pisa, Italy; giovanni.cilia@vet.unipi.it (G.C.); domenico.cerri@unipi.it (D.C.); filippo.fratini@unipi.it (F.F.)

**Keywords:** wild boar, intermediate *Leptospira*, *Leptospira fainei*, leptospirosis, reproductive system, testicles, epididymis, Italy, Tuscany region

## Abstract

**Simple Summary:**

Genital leptospirosis is an important example of the neglected infectious zoonotic disease caused by *Leptospira*. The disease was just evaluated in bovine and domestic pig with important consequences for reproductive success. Recently, pathogenic *Leptospira* strains were also isolated and detected from reproductive system tissues collected from wild boar (*Sus scrofa*) free ranging in the Tuscany and Sardinia regions (Italy). This investigation aimed to understand this aspect in wild boar, describing the detection of intermediate *Leptospira* DNA belonging to *Leptospira fainei* for the first time in male reproductive organs of hunted wild boar. The obtained data shed significant light on this intermediate *Leptospira* species, because, other than circulating in wildlife, it can localize in testicles and epididymides of wild boar specimens. These findings add important information on genital leptospirosis epidemiology, especially among the wildlife that remains less investigated.

**Abstract:**

Leptospirosis is a re-emerging and worldwide diffused zoonosis. Recently, the high importance of their epidemiology was explained by the intermediate *Leptospira* strains. Among these strains, *Leptospira fainei* was the first intermediate strain detected in domestic and wild swine. Wild boars (*Sus* *scrofa*) are well known as a reservoir, as well as all swine, for pathogenic *Leptospira*, but very little information is available concerning intermediate *Leptospira* infection. The investigation aim was to evaluate if intermediate *Leptospira* can infect the reproductive systems of wild boars hunted in the Tuscany region (Italy), as previously demonstrated for pathogenic ones. The reproductive system tissue (testicles, epididymides, uteri), and placentas and fetuses, were collected from 200 regularly hunted animals. Bacteriological examination and real-time PCR were performed to detect intermediate *Leptospira* DNA. Unfortunately, no isolates were obtained. Using real-time PCR, in six (3%) male organs (both testicles and epididymis), intermediate *Leptospira* DNA was found. The amplification of the *16S rRNA* gene identified that all DNA obtained belong to *Leptospira fainei.* The results of this investigation highlighted for the first time the localization of *Leptospira fainei* in the male wild boar reproductive system, opening up a new avenue to further investigate.

## 1. Introduction

Leptospirosis is a re-emerging and neglected zoonoses diffused in all parts of the world. The disease is caused by bacteria belonging to the order Spirochaetales and the genus *Leptospira* [1,2,3]. Recently, due to a genomic approach and based on their clinical presentation, the *Leptospira* species were divided into three groups: pathogenic, which cause the most severe infection; intermediate, which cause a mild to severe infection; and saprophytic, which are naturally present in the environment and do not usually cause disease [3,4,5,6]. Nowadays, the *Leptospira* genus includes 64 different species, with 17 belonging to pathogenic group (*Leptospira interrogans, Leptospira kirschneri, Leptospira noguchii, Leptospira borgpetersenii, Leptospira adleri, Leptospira alexanderi, Leptospira weilii, Leptospira santarosai, Leptospira kmetyi, Leptospira alstoni Leptospira mayottensis, Leptospira ellisii Leptospira barontonii, Leptospira dzianensis, Leptospira tipperaryensis, Leptospira gomenensis* and *Leptospira putramalaysiae*), 21 to the intermediate group (*Leptospira perolatii, Leptospira venezuelensis, Leptospira neocaledonica, Leptospira saintgironsiae, Leptospira haakeii, Leptospira hartskeelii, Leptospira licerasiae, Leptospira wolffii, Leptospira fainei, Leptospira broomii*, *Leptospira inadai, Leptospira andrefontaineae, Leptospira dzoumogneensis, Leptospira koniamboensis, Leptospira sarikeiensis, Leptospira johnsonii, Leptospira fluminis, Leptospira fletcheri, Leptospira semungkisensis, Leptospira langatensis* and *Leptospira selangorensis*), and 26 to the saprophytic group (*Leptospira idonii, Leptospira meyeri, Leptospira terpstrae, Leptospira biflexa, Leptospira levettiii, Leptospira brenneri, Leptospira harrisiae, Leptospira vanthielii, Leptospira yanagawae*, *Leptospira wolbachii, Leptospira bandrabouensis, Leptospira noumeaensis, Leptospira jelokensis, Leptospira bourretii, Leptospira kanakyensis, Leptospira kemamanensis, Leptospira mtsangambouensis, Leptospira bouyouniensis, Leptospira ellinghausenii, Leptospira congkakensis, Leptospira perdikensis, Leptospira montravelensis* and *Leptospira ognonensis, Leptospira ilyithenensis, Leptospira kobayashii, Leptospira ryugenii*) [3,7].

Due to its phylogenetic position, between saprophytic and pathogenic, the intermediate *Leptospira* species could have a wide range of antigens in common with pathogenic serovars, and they may be responsible for mild or severe infections [8]. Among intermediate species, *Leptospira fainei* play an important role in the epidemiology of leptospirosis. 

The intermediate *Leptospira fainei* serovar Hurstbridge was originally isolated, in 1994, from the kidneys and the uteri of domestic pigs bred in Australia [9], and, in the same year, was serologically detected in symptomatic and asymptomatic humans in Australia and the archipelagos of the Seychelles [10,11]. Later, leptospires belonging to this species were isolated from ill human patients with symptoms associable with leptospirosis in Denmark [12], France [13], Peru [14], India [5], and Ecuador [15].

Concerning animal *Leptospira fainei* infection, no isolates were obtained, but its DNA was detected in bats collected in Peru [16,17]. Consequently, within a leptospirosis survey, *Leptospira fainei* DNA was detected in wild boar (*Sus scrofa*) kidneys in Italy [18,19].

In Italy, as well as in all of Europe, wild boar is the most diffused wild ungulate [20,21]. This mammal is highly adaptable, and it can live in several habitats, especially near urban areas [20,22]. Wild boar is recognized as a reservoir of different *Leptospira* strains, especially for swine-adapted serogroups Tarassovi, Pomona and Bratislava [23,24,25,26,27,28,29,30]. In this animal species, as well as in all infected hosts, the main site of *Leptospira* localization is the kidney, with the consequent shedding of the bacteria in the environment via urine [1,2,31]. Moreover, a genital organ infection may occur a second time after bacteremia and/or renal localization [32,33,34]. However, recently, genital leptospirosis seems to be under consideration as a specific syndrome for bovine and swine [35]; indeed, genital leptospirosis has specific features, such as low systemic antibody titers, and chronic infection often associated in females with low fertility rates, abortion and perinatal mortality, and in males with testicular and epididymal infection [31,32,33,35,36,37,38]. In addition, sexual transmission was demonstrated [31,35]. In wild boar, genital infection was demonstrated, other than with the *Leptospira* DNA detection process undertaken in the reproductive system, through the isolation of the *Leptospira borgpetersenii* serogroup Ballum in aborted fetuses [39] and the isolation of *Leptospira interrogans* serovar Bratislava and *Leptospira kirschneri* serovar Grippotyphosa from testicles and epididymides [40].

This investigation aimed to evaluate the localization of intermediate *Leptospira* in the reproductive systems of male and female wild boar hunted in the Tuscany region (Italy).

## 2. Materials and Methods

### 2.1. Samples Collection

The samples employed in this investigation were the same as those previously reported [40], collected during the authorized hunting season 2018/2019 (October–January) according to the regional Italian hunting law (Regolamento di attuazione della legge regionale 12 gennaio 1994, n. 3 D.P.G.R. 48/R/2017—Regione Toscana). Briefly, from wild boar hunted in the Tuscany region, reproductive system tissue samples were collected, including testicles, epididymides, and uteri, as well as placentas and fetuses from pregnant wild boar. The fetuses from each pregnant wild boar were pooled and considered as a single sample. All organs were collected by veterinarians during the slaughtering activities, performed by hunters, minimizing all possible contamination [40]. Since sampling was conducted during the hunting season, the sample size could not be predicted beforehand. Moreover, for each specimen, sex and age were recorded. Age was determined after assessing the degree of tooth eruption and wear and tear of teeth of the lower jaw [41], subdividing them into young (under 12 months), subadult (between 12 and 24 months), and adult (over 24 months). No animals were specifically sacrificed for this study purpose.

### 2.2. Leptospira spp. Isolation

All organs were processed as previously reported [19,40]. Three distinct portions, approximately 1 cm^3^, of each tissue sample were homogenized into 5 mL of sterile phosphate-buffered saline (PBS) water using a Stomacher 400 Circulator (Seward LTD, West Sussex, United Kingdom). So, one milliliter of the homogenate was serially diluted in three tubes containing 5 mL of Ellinghausen–McCullough–Johnson–Harris (EMJH) media (Difco, Detroit, MI, USA), added with *Leptospira* enrichment EMJH (Difco) to perform the cultures. All tubes were then incubated at 30 ± 1 °C for 120 days and checked every 10 days through dark-field microscopy to evaluate possible bacterial growth. In case of contamination by other microorganisms, 2 mL from the contaminated tubes was filtered using 0.20 µm pore size filters and then sub-cultured in new EMJH media under the previously described conditions. Tubes were discarded if this procedure was not able to prevent further contamination.

### 2.3. Multiplex Real-Time PCR and Genotyping of Leptospira spp.

From each tissue samples, DNA was extracted using the Quick-DNA Plus Kit (Zymo Research, Irvine, CA, USA) following the manufacturer’s instructions. After the first multiplex real-time PCR performed to detect the *Leptospira* genus (*16S rRNA* gene) and pathogenic species (*lipL32* gene) [18], positive samples of *16S rRNA* only were analyzed using a second multiplex real-time PCR. The second protocol is based on intermediate *Leptospira* (*16S rRNA* gene) and saprophytic *Leptospira* (*23S rRNA* gene) using specific probes for each gene target [4,42]. The real-time PCR assay was done using the Rotorgene Corbett 6000 (Corbett Research, Sidney, Australia) with the following thermal conditions: a holding stage of 95 °C for 5 min, 45 cycles of 95 °C for 15 sec, and 60 °C for 30 sec. Samples were considered positive with a Ct < 40 [4,42]. DNA extracted from the strains employed as live antigens for MAT was used as the positive control. Sterile DNase- and RNase-free distilled water was employed as a negative control.

Finally, *Leptospira* species were identified using a primer for the *rrs2* gene from PCR-positive tissue samples [43]. The amplification of each target gene was performed using a HotStarTaq Master Mix Kit (Qiagen, Hilden, Germany). All amplicons were further sequenced (BMR Genomics, Padova, Italy) using the same amplification primer sets, and analyzed using BioEdit [44]. Phylogenetic analysis based on the *rrs2* gene was performed using the maximum likelihood method based on the Tamura–Nei model using MEGA 10 software [45].

### 2.4. Statistical Analysis

The obtained data were analyzed using Chi-square (*X*^2^) and Fisher (F) tests. Statistical tests were used to evaluate the *Leptospira* infection ratio regarding sex (male or female), age (young, subadult, or adult) and pregnancy (pregnant or not) of the wild boar. Besides this, stratified analyses were performed considering all the possible parameter combinations (i.e., only males stratified by age). The statistical significance threshold was set at a *p*-value ≤ 0.05 [46].

## 3. Results

During the hunting season, 200 wild boar specimens were sampled. Of them, 85 were males and 115 females (35 were pregnant), subdivided into 92 adults, 31 subadults, and 77 young animals. Moreover, of these, 75 were sampled in Grosseto province, 58 in Pisa province, 55 in Siena province, and 12 in Livorno province. No macroscopic lesions in the genital organs were detected in the post-mortem examination. Furthermore, no gross lesions were detected in other organs, and the animals were considered to be healthy.

No isolates of intermediate *Leptospira* or saprophytic *Leptospira* were obtained during the four months of culture incubation. Furthermore, all samples were negative for saprophytic *Leptospira* using the molecular method. On the other hand, intermediate *Leptospira* DNA was detected in six male organs (3%). For each male, both the testicles and epididymis were assessed as positive via real-time PCR. Of these positive males, three were adult and three young (Table 1).

Statistical difference was observed as regards sex, as intermediate *Leptospira* infection was shown to be significantly associated with males (*p* < 0.05). However, no statistical differences (*p* > 0.05) were recorded in the prevalence of intermediate *Leptospira* infection considering age and provinces.

**Figure 1:**
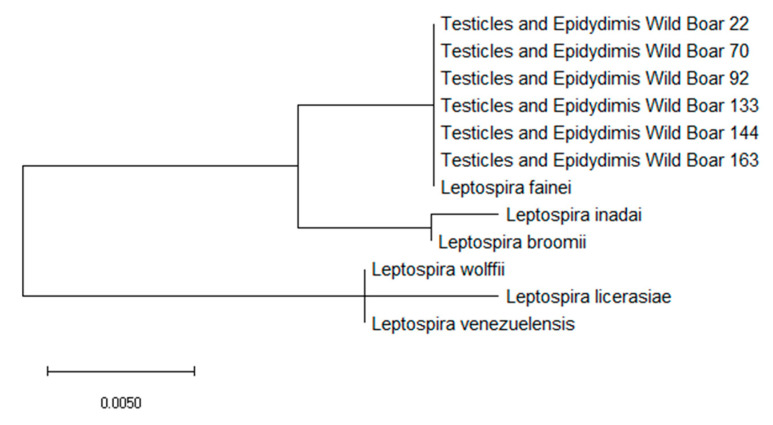
Molecular phylogenetic analysis for the *16s rRNA* gene of *Leptospira fainei*, *Leptospira inadai*, *Leptospira broomii*, *Leptospira wolfii*, *Leptospira licerasiae*, and *Leptospira venezuelensis* by the maximum likelihood method based on the Tamura–Nei model. The branch lengths of the tree measures the number of substitutions per site. The analysis involved 22 nucleotide sequences. There was a total of 438 positions in the final dataset.

Concerning the characterization of PCR-positive samples, the amplification of *16s rRNA* highlighted that intermediate *Leptospira* belonged to *Leptospira fainei*. The phylogenetic analysis identified a close relationship with this *Leptospira* species (Figure 1).

## 4. Discussion

In this study, leptospiral infection of the wild boar reproductive system, caused by intermediate *Leptospira*, was evaluated for the first time. Unfortunately, no isolates were obtained, but the DNA of intermediate *Leptospira* was detected. In particular, only samples from males of different age classes scored positively. According to phylogenetic analysis, the intermediate *Leptospira* DNA detected belonged to *Leptospira fainei.* Although no referable macroscopic lesions were observed during a post-mortem examination, the results of this investigation suggest that infection by *Leptospira fainei* in the genital organs could occur in males of different age classes.

Samples (kidney, liver, serum and reproductive organs) from the same animals were previously analyzed for *Leptospira* spp., and the results are already published [19,40]. In particular, considering only the wild boar found to be positive for *L. fainei* in the present investigation, all livers were negative for *Leptospira* spp., all kidneys and reproductive organs were negative for pathogenic leptospires, and all sera scored negatively for MAT. The isolation of *Leptospira* from all the organs failed, except for “wild boar 22”, from which the *L. interrogans* serogroup Australis serovar Bratislava (ST 24) was isolated.

Previously, only one study focused on *Leptospira* infection in the reproductive systems of wild boar has been carried out [40], but this is the first time that *Leptospira fainei* has been detected in testicles and epididymides. Moreover, in all positive males, both testicles and epididymides scored positively for *Leptospira*, suggesting a dual localization in both organs. As previously supposed by the same authors, the presence of *Leptospira*, pathogenic or intermediate, in the epididymis could be transitory as a consequence of infection at the testicular level [40], although no detailed information is available.

As reported for bull and domestic boar, genital leptospirosis is always subclinical. Although during the acute phases the disease could cause mild and temporary orchitis, the damage to spermatozoa and the influence on sperm viability remain unknown [31,32,35,47]. The venereal transmission of pathogenic *Leptospira* could occur in swine, as well as in other animals like cattle, via infected semen that could carry the pathogen in the female uterus and oviduct [35,48,49,50]. Therefore, it is presumable that *Leptospira fainei* could be transmitted from males to females during coitus. However, no female reproductive organs scored positively. A possible explanation for this could be that this strain could not be adapted to the uterine environment of wild boar. Another possible hypothesis is that males could act as asymptomatic carriers, while females are susceptible to infection that evolves into clinical manifestations. This hypothesis seems to be supported by the fact that the detection of *L. fainei* DNA was statistically associated with males, but it is difficult to fully demonstrate due to the impossibility of detecting symptoms or reproductive disorders in wild animals. Nevertheless, it is important to stress that *Leptospira fainei* was initially isolated from the uteri of domestic pigs in Australia, suggesting a tropism for the female swine genital system, although information on the associated disease is scant [9]. The infection in the genital tract of sows could represent a risk for domestic animals due to the reproductive behavior of wild boar males that often mate with sow breeds in extensive or semi-extensive farms [51]. Finally, the obtained data (zero positivity among fetuses, placentas and pregnant uteri) suggest a low tropism of *Leptospira fainei* for wild boar fetuses and fetal annexes, but a vertical transmission cannot be excluded with certainty.

The different results obtained between cultural and molecular methods could be partially due to the medium employed. Indeed, nEMJH medium was used in this investigation, while in other studies aimed at isolating intermediate *Leptospira*, EMJH medium added with STAFF supplement containing five antimicrobials (sulfamethoxazole, trimethoprim, amphotericin B, fosfomycin, and 5-fluorouracil) was employed [3,4,5]. However, STAFF supplement makes the medium more selective, but not more suitable for *Leptospira*; it only helps to prevent contamination. Furthermore, *L. fainei* was isolated by other authors with conventional EMJH without modifications. In light of these considerations, although the use of the STAFF supplement could probably enhance the opportunities to isolate intermediate strains, the use of classical medium did not fully preclude the possibility of obtaining isolates; indeed, the same method allowed the authors to isolate pathogenic *Leptospira* strains from the same samples [19,40].

Only very few studies have highlighted *Leptospira fainei* infection in swine. In Italy, it was previously detected in the kidneys of wild boar collected in the Tuscany and, presumably, Liguria regions [18,19]. The prevalence of intermediate *Leptospira* recorded in this investigation (3%) was very similar to that reported in wild boar kidneys from the Tuscany and Liguria regions [18,19]. In particular, the kidneys and livers from the same animals analyzed in this investigation were previously tested by PCR; all livers were negative, while 9/287 (3.14%) of the kidneys were positive for intermediate *Leptospira* [19]. However, the animals that were positive at the kidney level were negative at the reproductive level and vice versa. The observed lack of correlation between *Leptospira fainei* infection in the kidney and genital tracts suggests two possible specific independent transmission routes for this intermediate strain: one venereal and one conventional via contaminated urines. 

The impact of *Leptospira fainei* infection on the reproductive systems of male wild boar is of great interest because among neglected diseases such as leptospirosis, infection by intermediate strains is highly underestimated. Furthermore, it could potentially have an impact on population breeding. Indeed, in the natural ecosystem, control of genital leptospirosis and its impact on the reproductive performance of wildlife, as well as in wild boar, is very difficult to maintain.

Finally, *Leptospira fainei* infections in the reproductive systems of wild boar may also represent a risk for human health, especially for hunters. Based on the isolation and detection of *Leptospira fainei* infection in humans [5,12,13,15,16], the risk for hunters is represented by exposure to the pathogen during the slaughter activities; indeed, the reproductive systems are necessarily handled, commonly without the use of personal protective equipment.

## 5. Conclusions

In concluding, the results of this investigation suggest that the genital tracts of male wild boar could be a target for *Leptospira fainei*, as well as for different *Leptospira interrogans* and *Leptospira kirschneri* [40].

Genital *Leptospira* infection could be linked to reproductive complications, as recently evidenced in the domestic pig. It is likely that the wild boar is more resistant than selected breeding lineages, and this species is not exposed to intensive farming stress [40]. For these reasons, the impact of genital *Leptospira* infection on the reproductive performance might be lower than in domestic swine, as suggested by the high birth rates reported recently in Italy, as well in Tuscany [21,52].

Furthermore, the localization of *Leptospira fainei* in the male genital organs could represent a public health risk, mainly for the hunters and stakeholders that commonly handle these organs without personal protective equipment, increasing the contamination and infection.

Finally, the localization of *Leptospira fainei* suggests a new scenario within the epidemiology of leptospirosis in wild boar. More investigations must be carried out to understand the occurrence of intermediate leptospirosis in the reproductive system, the implications of genital leptospirosis for reproductive performance, and the possibility of venereal transmission from males to females and its consequences.

## Figures and Tables

**Table 1:** Provinces and age classes of *Leptospira fainei-*positive wild boar sampled in the Tuscany region.

Samples	Province	Age Class	*Leptospira* Species
Wild Boar 22	Livorno	Young	*Leptospira fainei*
Wild Boar 70	Grosseto	Adult	*Leptospira fainei*
Wild Boar 92	Pisa	Young	*Leptospira fainei*
Wild Boar 133	Grosseto	Adult	*Leptospira fainei*
Wild Boar 144	Pisa	Young	*Leptospira fainei*
Wild Boar 163	Grosseto	Adult	*Leptospira fainei*

## Data Availability

Not applicable.
